# Comparison of advanced echocardiographic right ventricular functional parameters with cardiovascular magnetic resonance in adult congenital heart disease

**DOI:** 10.1093/ehjimp/qyad033

**Published:** 2023-10-11

**Authors:** Daniel J Bowen, Robert M Kauling, Chiara Pelosi, Lourus van Haveren, Jackie S McGhie, Judith A A E Cuypers, Alexander Hirsch, Jolien W Roos-Hesselink, Annemien E van den Bosch

**Affiliations:** Department of Cardiology, Erasmus MC University Medical Center Rotterdam, Dr. Molewaterplein 40, 3015 GD Rotterdam, The Netherlands; Department of Cardiology, Erasmus MC University Medical Center Rotterdam, Dr. Molewaterplein 40, 3015 GD Rotterdam, The Netherlands; Department of Cardiology, Erasmus MC University Medical Center Rotterdam, Dr. Molewaterplein 40, 3015 GD Rotterdam, The Netherlands; Department of Cardiology, Erasmus MC University Medical Center Rotterdam, Dr. Molewaterplein 40, 3015 GD Rotterdam, The Netherlands; Department of Cardiology, Erasmus MC University Medical Center Rotterdam, Dr. Molewaterplein 40, 3015 GD Rotterdam, The Netherlands; Department of Cardiology, Erasmus MC University Medical Center Rotterdam, Dr. Molewaterplein 40, 3015 GD Rotterdam, The Netherlands; Department of Cardiology, Erasmus MC University Medical Center Rotterdam, Dr. Molewaterplein 40, 3015 GD Rotterdam, The Netherlands; Department of Radiology and Nuclear Medicine, Erasmus MC University Medical Center Rotterdam, Rotterdam, The Netherlands; Department of Cardiology, Erasmus MC University Medical Center Rotterdam, Dr. Molewaterplein 40, 3015 GD Rotterdam, The Netherlands; Department of Cardiology, Erasmus MC University Medical Center Rotterdam, Dr. Molewaterplein 40, 3015 GD Rotterdam, The Netherlands

**Keywords:** 3D echocardiography, multi-plane echocardiography, cardiovascular magnetic resonance, right ventricular longitudinal strain, speckle tracking, feature tracking

## Abstract

**Aims:**

Advanced transthoracic echocardiography (TTE) using volumetric and deformational indices provides detailed quantification of right ventricular (RV) function in adults with congenital heart disease (ACHD). Two-dimensional multi-plane echocardiography (2D-MPE) has demonstrated regional wall differences in RV longitudinal strain (LS). This study aims to evaluate the association of these parameters with cardiovascular magnetic resonance (CMR).

**Methods and results:**

One-hundred stable ACHD patients with primarily affected RVs were included (age 50 ± 5 years; 53% male). Conventional and advanced echocardiographic RV functional parameters were compared with CMR-derived RV function. Advanced echocardiographic RV functional parameters were measurable in approximately one-half of the study cohort, while multi-wall LS assessment feasibility was lower. CMR RV ejection fraction (CMR-RVEF) was moderately correlated with deformational, area, and volumetric parameters [RV global LS (lateral wall and septum), *n* = 55: *r* = −0.62, *P* < 0.001; RV wall average LS, *n* = 34: *r* = −0.49, *P* = 0.002; RV lateral wall LS, *n* = 56: *r* = −0.45, *P* < 0.001; fractional area change, *n* = 67: *r* = 0.48, *P* < 0.001; 3D-RVEF, *n* = 48: *r* = 0.40, *P* = 0.005]. Conventional measurements such as TAPSE and RV S′ correlated poorly. RV global LS best identified CMR-RVEF < 45% (area under the curve: 0.84, *P* < 0.001: cut-off value −19%: sensitivity 100%, specificity 57%). RVEF and LS values were significantly higher when measured by CMR compared with TTE (mean difference RVEF: 5 [−9 to 18] %; lateral (free) wall LS: −7 [7 to −21] %; RV global LS: −6 [5 to −16] %) while there was no association between respective LS values.

**Conclusion:**

In ACHD patients, advanced echocardiographic RV functional parameters are moderately correlated with CMR-RVEF, although significant differences exist between indices measurable by both modalities.

## Graphical Abstract

**Graphical abstract:**
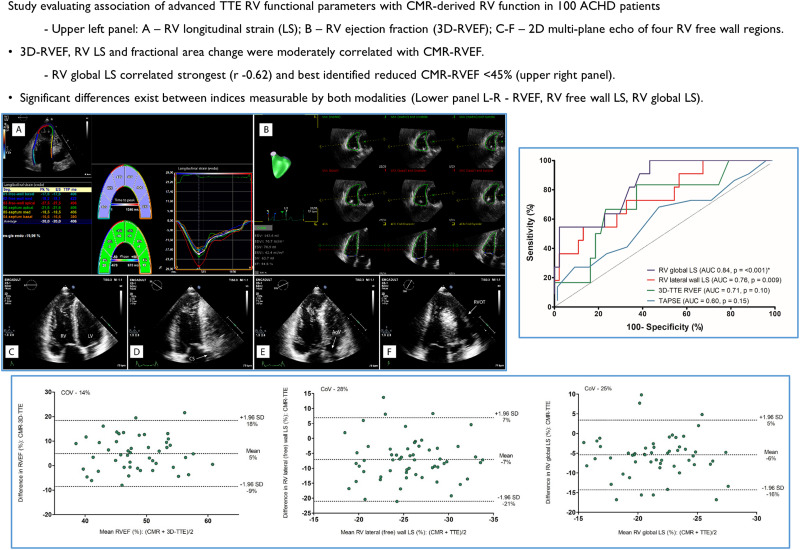

## Introduction

Evaluation of right ventricular (RV) function by transthoracic echocardiography (TTE) can often be challenging in adults with congenital heart disease (ACHD), particularly in instances of altered cardiovascular and musculoskeletal anatomy or following multiple surgical interventions.^1^ While conventional indices that evaluate RV longitudinal shortening are highly feasible and reproducible, the addition of advanced deformational or volumetric parameters is preferable to enhance RV functional assessment.^2^ As the global population of ACHD patients continues to grow and age, the dependency on cardiovascular magnetic resonance (CMR) to provide accurate assessment of RV function will increase. However, as a more accessible modality, echocardiography has an important role to play in reducing the burden on CMR.^3^ For ACHD patients with good to reasonable echocardiographic image quality, it is important to define which functional measurements demonstrate an acceptable level of agreement with CMR. Several studies have investigated the association between conventional and advanced echocardiographic parameters and CMR in ACHD populations.^1,4–7^ In this study, we also include two-dimensional multi-plane echocardiographic (2D-MPE) imaging, which enables quantitative assessment of four different RV free wall regions from one apical acoustic window using electronic plane rotation.^8^ We previously reported high feasibility for quantification of RV function with 2D-MPE in ACHD populations and provided new insights into regional RV wall deformation.^9,10^ The performance of regional RV wall deformation compared with CMR has however not yet been demonstrated. Furthermore, with the emergence of CMR feature tracking (CMR-FT),^11^ it is of interest to investigate how comparable longitudinal strain (LS) measurements are with speckle tracking echocardiography (STE) in this patient population. This study therefore aims to evaluate the association of these parameters, alongside other conventional and advanced echocardiographic indices with reference CMR-derived RV function in ACHD.

## Methods

### Study population

The study population consists of ACHD patients who participated in the Quality of Life 4 study at the Erasmus Medical Center (EMC) in Rotterdam, between February 2020 and September 2021. The Quality of Life study was initiated by EMC in 1990 and is performed every 10 years. The study follows up individuals born with ACHD whose primary surgical repair took place before the age of 15 years old between 1968 and 1980. For this study, only individuals with initial pathologies primarily affecting the RV were included, diagnoses were atrium septum defect (ASD), Tetralogy of Fallot (ToF), and pulmonary stenosis (PS). Subjects were excluded from analysis if CMR and TTE were not performed on the same day. The study was carried out according to the principles of the Declaration of Helsinki, was approved by the local medical ethics committee (MEC-2019 0465), and written informed consent was obtained from all subjects.

### Echocardiographic acquisition and conventional measurements

An extensive TTE protocol was carried out according to international guidelines^12^ with additional focus on RV structure and function by acquiring 2D-MPE and 3D-TTE recordings in individuals where image quality permitted. All echocardiograms were performed by one of two echocardiographers (D.J.B., L.v.H.) specialized in congenital echocardiography. Studies were acquired using an EPIQ7 ultrasound system (Philips Medical Systems, Best, The Netherlands) equipped with an X5-1 matrix array transducer (composed of 3040 elements with 1–5 MHz). Single beat 3D recordings of the right heart were acquired using Heart Model software (Philips Medical Systems). Conventional 2D echocardiographic parameters for left and right heart size and function were collected in addition to the grading of any valvular lesions as either less than (<) or equal or greater than (≥) moderate in severity using parameters as documented in published guidelines.^13,14^ RV basal, mid, and longitudinal linear dimensions alongside fractional area change (FAC, calculated as end-diastolic area − end-systolic area/end-diastolic area × 100) were measured in the standard focused RV apical four-chamber view. Tricuspid annular plane systolic excursion (TAPSE) and tissue Doppler imaging derived tricuspid annular peak systolic velocity (RV S′) were measured at the basal lateral RV wall.

### Advanced right ventricular assessment by 2D multi-plane and 3D echocardiography

**Figure 1:**
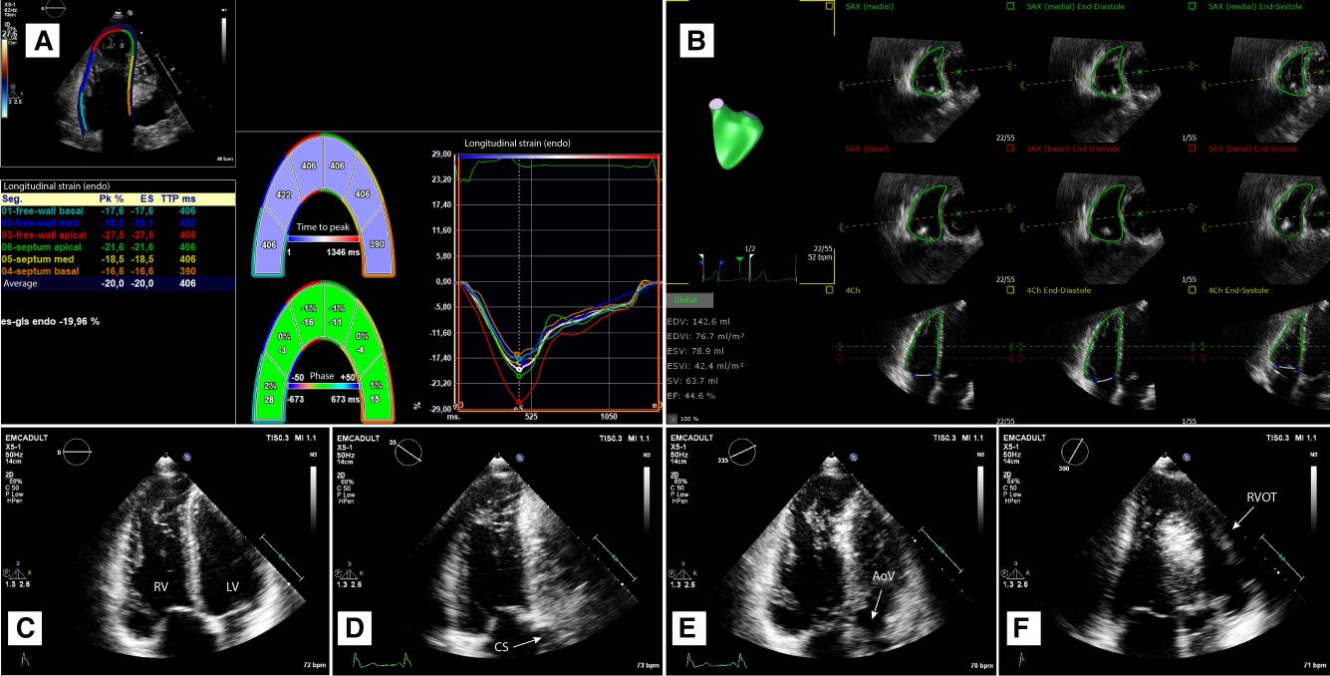
Advanced echocardiographic imaging for the assessment of RV function. Top left panel (*A*)—right ventricular longitudinal strain. Top right panel (*B*)—3D right ventricular ejection fraction. Lower panels (from left to right)—2D multi-plane echocardiography (approximate degrees of electronic rotation, RV wall visualized): C—RV-focused apical four-chamber view (0°, lateral wall); D—coronary sinus view (+40°, anterior wall); E—aortic view (−40°, inferior wall); F—coronal view (−90°, inferior wall and RVOT). Adapted from Bowen *et al*.^9^ with permission. RV, right ventricle; LV, left ventricle; CS, coronary sinus; AoV, aortic valve; RVOT, right ventricular outflow tract.

The evaluation of regional RV wall function by 2D multi-plane echocardiography has been well documented in our previous publications.^8,9^ In short, from a fixed apical probe position, electronic plane rotation around the RV apex allows visualization of different RV free wall regions. Each RV wall is confirmed by the presence of a certain left-sided landmark associated with an approximate degree of electronic rotation. The first view at 0˚ shows the lateral RV wall with the left-sided landmark being the mitral valve. The second view at ∼+40˚ shows the anterior RV wall and the coronary sinus, thirdly at ∼−40˚ the inferior RV wall and the aortic valve, and lastly at ∼−90˚ the inferior coronal view with the inferior wall and the anterior segment of the RV outflow tract (RVOT) (*Figure 1*). The four RV wall datasets were digitally exported to a vendor-neutral server (TomTec Imaging Systems, Unterschleissheim, Germany), and data analysis was performed offline by one independent observer (DB) using DICOM greyscale images. To assess peak systolic RV LS, an RV algorithm wall motion tracking software was used (2D CPA, Image-Arena version 4.6; TomTec Imaging Systems). The endocardial border of the RV free wall and septum were manually traced at end-systole and adjusted accordingly in end-diastole if required. This was performed in each of the four multi-plane views previously described. A single segment peak LS value for each RV wall was derived from the average of the basal, mid, and apical segments. Global LS was calculated by averaging the strain values of the lateral wall and inter-ventricular septum. An RV wall average value was calculated when LS of the lateral, anterior, and inferior walls were all feasible to measure in an individual. The 3D datasets were digitally exported to the same TomTec server and analysed retrospectively by DB using specialized RV analysis software (TomTec 4D-RV function 2.0). After placing set landmarks, RV volumes (indexed for body surface area) and ejection fraction (RVEF) were automatically calculated over the entire cardiac cycle. All contours were checked and manually adjusted when necessary.

### Cardiovascular magnetic resonance

CMR examinations were performed on a clinical 1.5T MRI system (SIGNA Artist, GE Healthcare, Milwaukee, WI, USA) with a dedicated cardiac or anterior array coil, electrocardiographic gating, and breath-hold techniques. Standard balanced steady-state free precession (bSSFP) cine images were obtained during end-expiratory breath-hold in standard three long-axis views (two-, three-, and four-chambers) and in a contiguous stack of short-axis (SA) views, with coverage from base to apex. bSSFP scan parameters were as follows: slice thickness long-axis 8 mm and SA 6 mm, interslice gap 4 mm, TR/TE 3.8/1.7 ms, flip angle 65◦, ASSET 2, acquired matrix long-axis 280 × 200 and SA 200 × 200, and 30 phases per cardiac cycle. CMR analysis was performed on anonymized images by an experienced CMR reader (J.A.A.E.C.). Functional analysis was performed on SA images using automatic segmentation of the epi- and endocardial contours in end-systolic and end-diastolic phase, with inclusion of papillary muscles and trabeculations in the left ventricular and RV volume. All contours were checked and manually adjusted when necessary. Endocardial RV LS was measured in the four-chamber long axis using CMR-FT. The RV endocardial contours were drawn manually during the end-diastolic and end-systolic phase by one operator (D.J.B.). Subsequently, the software automatically traced the cardiac contours during the cardiac cycle, resulting in the calculation of peak LS of the RV free wall and inter-ventricular septum. Global LS was calculated as the average of these two values as per TTE. CMR analyses were performed using commercially available software (Qmass version 8.1 and QStrain version 4.0, Medis Medical Imaging Systems, Leiden, The Netherlands). All analyses were performed blinded to the results of the other imaging modality.

### Statistical analysis

The distribution of data was assessed using histograms and the Shapiro–Wilk test. Continuous data are presented as mean ± standard deviation (SD) or median [inter-quartile range], while categorical data are presented as frequencies. The paired *t*-test or Wilcoxon signed rank test was used for comparison of within-subject LS values and for RV functional parameters measurable by both TTE and CMR. Linear regression analysis was performed to evaluate the association between echocardiographic RV functional parameters and CMR-derived RVEF, in addition to LS values measured by both modalities [RV lateral (free) wall and RV global LS]. Pearson’s correlation coefficient (*r*) is reported and interpreted as follows: <0.2 = poor, 0.20–0.39 = fair, 0.40–0.59 = moderate, 0.60–0.79 = good, and 0.80–1.00 = excellent. Receiver operating characteristic (ROC) curves were created to determine the ability of echocardiographic parameters to detect reduced CMR-RVEF (cut-off value of 45% used^15^). The area under the curve (AUC) is reported in addition to the ‘optimal’ cut-off value for each parameter to detect reduced RVEF. This is defined as the value that corresponds with the highest Youden-J index (specificity + sensitivity − 1), whereby a value of 1 indicates perfect detection and a value of 0 no detection. Agreement between CMR-derived and TTE-derived measurements was evaluated using Bland–Altman analysis.^16^ The agreement between two measurements was determined as the mean of the differences +1.96 times their SD. Additionally, the coefficient of variation was provided (SD of the differences of two measurements divided by their mean value, times 100). All statistical analyses were performed using the Statistical Package for Social Sciences version 25 (SPSS, Inc., Armonk, NY, USA). The statistical tests were two-sided and a *P*-value of <0.05 was considered statistically significant.

## Results

**Figure 2:**
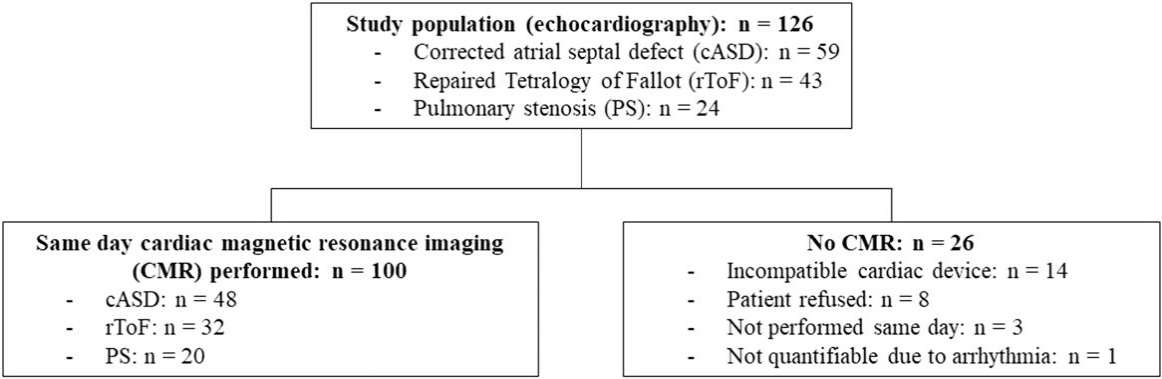
Study inclusion.

One-hundred ACHD patients (age 50 ± 5 years, 53% male) were included (*Figure 2*), all with initial pathologies primarily affecting the RV (ASD *n* = 48; ToF *n* = 32; PS *n* = 20). Age at definitive surgical correction was 6 [3, 8] years, while 25 individuals had undergone surgical re-intervention. Demographics, electrocardiogram and echocardiographic characteristics are detailed in *Table 1*. Very few patients had ≥moderate right-sided valve disease: pulmonary valve insufficiency—in 11 patients; pulmonary valve stenosis—in 8; and tricuspid regurgitation—in 5. Only one individual had significantly reduced left ventricular systolic function, and five individuals had high grade (II/III) diastolic dysfunction.

**Table 1:** Baseline characteristics and transthoracic echocardiography

	ACHD patients (*n* = 100)
*Demographics*	
Age, years	50 ± 5
Sex (male), *n*	53
Body mass index, kg/m²	26.5 ± 4.6
Systolic blood pressure, mmHg	132 ± 16
Diastolic blood pressure, mmHg	82 ± 12
*Electrocardiogram*	
Sinus rhythm, *n*	94
Atrial rhythm, *n*	6
QRS duration, ms	109 [95, 126]
*Congenital group and cardiothoracic intervention*	
Corrected atrial septal defect, *n*	48
Repaired Tetralogy of Fallot, *n*	32
Pulmonary stenosis, *n*	20
Age at definitive surgical correction, years	6 [3, 8]
Surgical re-intervention, *n*	25
*Transthoracic echocardiography*	
Right ventricular basal dimension, mm (*n* = 90)	44 [40, 49]
Right ventricular mid dimension, mm (*n* = 79)	33 [30, 38]
Right ventricular longitudinal dimension, mm (*n* = 86)	86 ± 10
Right ventricular outflow tract 1 dimension, mm (*n* = 65)	38 ± 5
3D RV end-diastolic volume index, mL/m²	74 [61, 88]
3D RV end-systolic volume index, mL/m²	38 [33, 50]
Right atrial area, cm² (*n* = 86)	19 [16, 22]
≥Moderate pulmonary regurgitation, *n*	11
≥Moderate pulmonary stenosis, *n*	8
≥Moderate tricuspid regurgitation, *n*	5
Tricuspid regurgitation maximum velocity, m/s (*n* = 83)	2.4 [2.2, 2.6]
Systolic pulmonary artery pressure, mmHg (*n* = 83)	28 [24, 34]
Pulmonary valve peak gradient, mmHg (*n* = 99)	9 [5, 18]
Left heart function, *n* (%):	
Significantly impaired systolic function (LVEF ≤ 45%)	1
Grade II–III diastolic function	5
≥Moderate left-sided valvular disease	0

Data are presented as mean ± standard deviation, as median [25th, 75th percentile], or number.

RV, right ventricle; LVEF, left ventricular ejection fraction.

### Measurement feasibility

**Figure 3:**
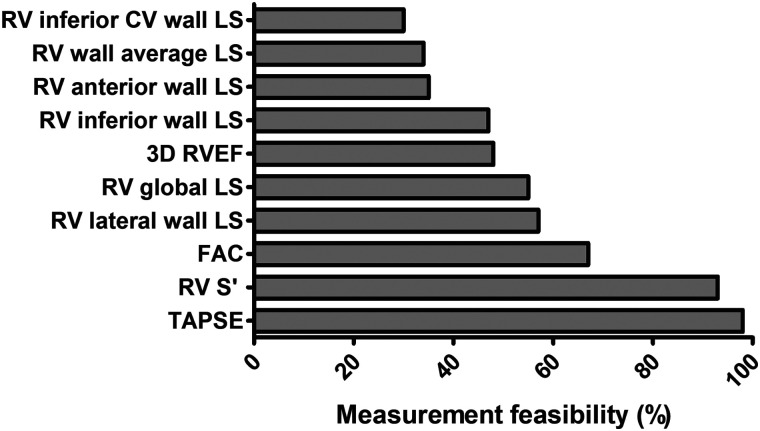
Feasibility of echocardiographic right ventricular functional parameters. TAPSE, tricuspid annular plane systolic excursion; RV S′, tricuspid annular peak systolic velocity; FAC, fractional area change; LS, longitudinal strain; RV global LS, right ventricular global longitudinal strain (lateral wall and septum); RVEF, right ventricular ejection fraction; RV wall average LS, lateral, anterior, and inferior wall LS all feasible.

Measurements of all echocardiographic functional indices were attempted in all patients where image quality permitted. The measurement feasibility of each parameter is demonstrated in *Figure 3*. Conventional parameters were the most feasible to perform (TAPSE in 98 patients; RV S′ in 93; FAC in 67). RV lateral wall and global LS were the most feasible deformational parameters (in 57 and 55, respectively) and more performable than 3D-RVEF (in 48). LS measurement feasibility was lower in the other RV walls with three walls measurable in 34 individuals. CMR volumetric measurements were performed in all patients. LS measurements using CMR-FT were performed in 98 patients of which 56 RV lateral (free) wall and 55 RV global LS values were comparable with those of STE. All echocardiographic parameters are demonstrated in *Table 2*, with those of CMR in *Table 3*. RV global LS was significantly lower than RV lateral wall LS (−19 ± 4% vs. −22 ± 5%, *P* < 0.001). Differences across the RV walls were evident, although only inferior coronal view wall LS was significantly lower than lateral wall LS (−19 ± 4%, *P* = 0.004).

**Table 2:** Associations of echocardiographic right ventricular functional parameters with cardiovascular magnetic resonance derived right ventricular ejection fraction

	No. comparisons	TTE values	CMR values	*r*	*P*-value
*RV two-dimensional conventional echo*					
TAPSE, mm	98	17 ± 3		0.16	0.06
RV S′, cm/s	93	10 ± 2		0.23	**0**.**015**
Fractional area change, %	67	38 ± 7		0.48	**<0**.**001**
*RV three dimensional echo*					
3D RV ejection fraction, %	48	46 ± 6	51 ± 6	0.40	**0**.**005**
*RV multi-plane echo longitudinal strain (LS), %*					
Global LS (lateral wall and septum)	55	−18 ± 5	−24 ± 4	−0.62	**<0**.**001**
Lateral (free) wall LS	56	−22 ± 5	−29 ± 5	−0.45	**<0**.**001**
Anterior wall LS	35	−20 ± 4		−0.41	**0**.**007**
Inferior wall LS	47	−22 ± 4		−0.22	0.07
Inferior coronal view wall LS	30	−19 ± 4*		−0.08	0.34
RV wall average LS	34	−21 ± 4		−0.49	**0**.**002**

Data are presented as mean ± standard deviation with *r*- and *P*-values reflecting correlation of TTE values with CMR-RVEF. Values in bold indicate a *P*-value <0.05. RV wall average LS calculated when lateral, anterior, and inferior walls all feasible to measure.

RV, right ventricle; TAPSE, tricuspid annular plane systolic excursion; RV S′, tissue Doppler imaging derived tricuspid annular peak systolic velocity.

^*^P < 0.01 vs. RV lateral wall LS.

**Table 3:** Right ventricular functional analysis by cardiovascular magnetic resonance

	All ACHD patients	Patients with feasible TTE measurements^a^	*P*-value
Volumetric (*n* = 100)			
RV end-diastolic volume index, mL/m²	98 [82, 117]	100 [86, 119]	0.49
RV end-systolic volume index, mL/m²	47 [39, 61]	49 [40, 60]	0.84
RV ejection fraction, %	50 ± 8	51 ± 6	0.49
Feature tracking (*n* = 98)			
RV free wall longitudinal strain, %	−30 ± 5	−29 ± 5	0.61
RV global longitudinal strain, %	−25 ± 4	−24 ± 4	0.73

Data are presented as mean ± standard deviation or as median [25th, 75th percentile].

RV, right ventricle.

^a^Volumetric data *n* = 48; RV free wall longitudinal strain *n* = 56; RV global longitudinal strain *n* = 55.

### Comparison with CMR

**Figure 4:**
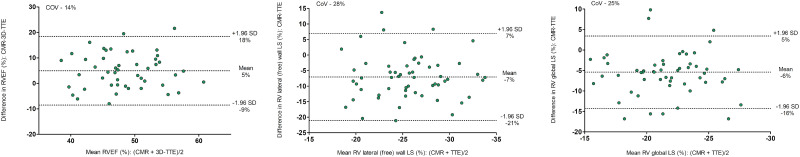
Bland–Altman plots demonstrating agreement between right ventricular functional parameters derived by TTE and CMR. Left panel—right ventricular ejection fraction (RVEF); centre panel—RV lateral (free) wall longitudinal strain; right panel—RV global LS (lateral wall and septum).

The associations between CMR-RVEF and the best performing echocardiographic functional parameters were moderate (*Table 2*). 3D-TTE-derived RV volumes and RVEF were significantly lower than those derived by CMR (RV end-diastolic volume index [RVEDVi]: 74 [61, 88] mL/m² vs. 100 [86, 119] mL/m², *P* < 0.001; RV end-systolic volume index [RVESVi]: 38 [33, 50] mL/m² vs. 49 [40, 60] mL, *P* < 0.001; RVEF 46 ± 6% vs. 51 ± 6%, *P* < 0.001). The association between 3D-RVEF and CMR-RVEF was moderate (*r* = 0.40, *P* = 0.005) with a mean difference of 5 [−9 to 18] % between respective measurements [coefficient of variation (CoV) −14%, *Figure 4*]. Of the deformational parameters, global LS (*r* = −0.62, *P* < 0.001), lateral wall LS (*r* = −0.45, *P* < 0.001), anterior wall LS (*r* = −0.41, *P* = 0.007), and RV wall average LS (*r* = −0.49, *P* = 0.002) values correlated strongest with CMR-RVEF. Of the conventional parameters, FAC correlated strongest with CMR-RVEF (*r* = 0.48, *P* < 0.001) however there was no association with TAPSE (*r* = 0.16, *P* = 0.06) or RV S′ (*r* = 0.23, *P* = 0.23).

RV LS values were significantly higher when measured by CMR-FT than by STE [lateral (free) wall LS: −29 ± 5% vs. −22 ± 5%, *P* < 0.001; global LS −24% ± 4% vs. −18 ± 5, *P* < 0.001]. There was a mean difference of −7 [7 to −21] % between lateral (free) wall LS measurements (CoV—28%) and −6 [5 to −16] % between global LS measurements (CoV—25%). Furthermore, there was no association between respective strain values [lateral (free) wall LS: *r* = 0.12, *P* = 0.37; global LS: *r* = 0.09, *P* = 0.49].

**Figure 5:**
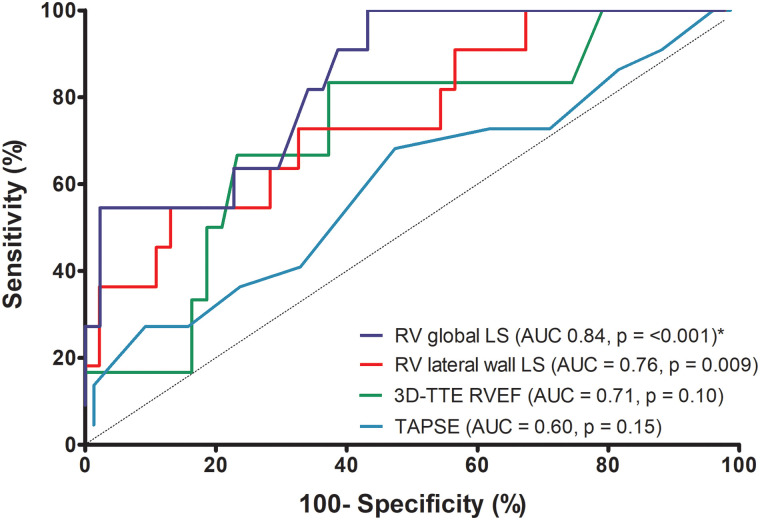
Receiver operating characteristic curves displaying the ability of right ventricular echocardiographic functional parameters to identify diminished CMR-derived right ventricular ejection fraction (RVEF < 45%). AUC, area under the curve. *P < 0.05 for RV global LS vs. RV lateral wall LS and RV global LS vs. TAPSE.

RV dysfunction was identified in 23 patients using the criteria of a CMR-RVEF < 45%. ROC curve analysis (*Table 4*, *Figure 5*) revealed RV global LS to be the best identifier of CMR-RVEF <45% (AUC: 0.84 [0.72–0.96], *P* < 0.001: cut-off value of −19%: sensitivity 100%, specificity 57%), with statistical significance compared with RV lateral wall LS (AUC: 0.76 [0.60–0.92], *P* = 0.04), TAPSE (AUC: 0.60 [0.46–0.74], *P* = 0.03), and FAC (AUC: 0.68 [0.52–0.83], *P* = 0.01). In the context of a low number of observations, differences between the AUC of other parameters were not statistically significant (*P* > 0.05).

**Table 4:** Receiver operating characteristics and optimal cut-off values of echocardiographic parameters to identify reduced right ventricular ejection fraction (<45%) by cardiovascular magnetic resonance

	AUC (95% CI)	*P*-value	Cut-off	Sens.	Spec.	Youden-J index
TAPSE (*n* = 98)	0.60 (0.46–0.74)*	0.15	18	68	53	0.21
RV S′ (*n* = 93)	0.63 (0.49–0.77)	0.07	11	76	36	0.12
Fractional area change (*n* = 67)	0.68 (0.52–0.83)*	**0**.**045**	35	57	76	0.33
3D right ventricular ejection fraction (*n* = 48)	0.71 (0.49–0.93)	0.10	44	83	62	0.45
Global LS (lateral wall and septum, *n* = 55)	0.84 (0.72–0.96)	**<0**.**001**	−19	100	57	0.57
Lateral wall LS (*n* = 56)	0.76 (0.60–0.92)*	**0**.**009**	−21	73	67	0.40
Anterior wall LS (*n* = 35)	0.81 (0.63–0.98)	**0**.**010**	−19	88	78	0.65
Inferior wall LS (*n* = 47)	0.67 (0.48–0.86)*	0.10	−20	70	57	0.27
Inferior coronal view wall LS (*n* = 30)	0.60 (0.37–0.83)	0.40	−19	67	71	0.38
RV wall average LS (*n* = 34)	0.82 (0.66–0.98)	**0**.**007**	−21	88	65	0.53

Cut-off value used in receiver operating characteristics analysis defined as CMR-derived RVEF of 45%. Values in bold indicate a *P*-value <0.05. RV wall average LS calculated when lateral, anterior, and inferior walls all feasible to measure.

TAPSE, tricuspid annular plane systolic excursion; RV S′, tissue Doppler imaging derived tricuspid annular peak systolic velocity; LS, longitudinal strain; AUC, area under the curve; CI, confidence interval.

^*^P < 0.05 for AUC vs. global LS.

## Discussion

**Figure 6:**
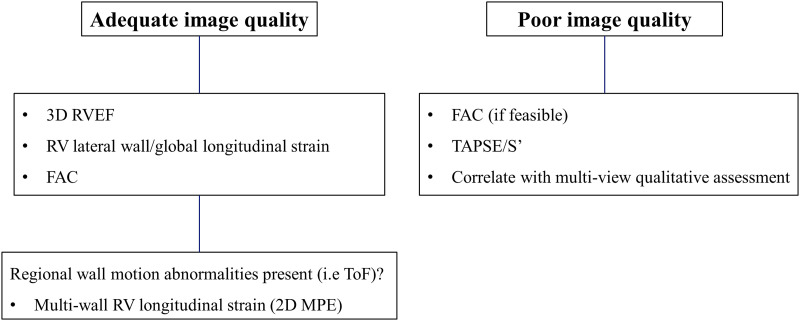
Suggested approach to right ventricular functional assessment by transthoracic echocardiography in ACHD patients.

Accurate assessment of RV function is essential when following up ACHD patients, with echocardiography the first line imaging modality available to the cardiologist. In recent years, advances in ultrasound probe technology and quantification software have opened up new possibilities for the evaluation of RV function. In this study of ACHD patients, advanced echocardiographic RV functional indices such as 3D-RVEF and RV LS were moderately correlated with reference CMR-derived RVEF. However, significant differences were observed between indices measurable by both modalities. Conventional FAC provided a comparable representation of CMR-RVEF to that of 3D-RVEF and RV LS, and was more feasible to perform. While highly feasible, TAPSE and S′ measurements correlate poorly with CMR-RVEF and should not be used in isolation to evaluate RV function in ACHD patients. LS averaged across multiple RV walls did not associate significantly better with CMR-RVEF than the global or lateral (free) wall values. 2D-MPE may only provide additional functional information when notable regional wall motion abnormalities are present, such as in ToF (*Figure 6*).

### Conventional echocardiographic parameters

Conventional TTE parameters that assess longitudinal function at the basal RV inlet (TAPSE, RV S′) correlated poorly with CMR-RVEF. In ACHD patients, these measurements do not represent global RV function if regional abnormalities are present.^17^ Furthermore, basal RV longitudinal contraction in post-operative ACHD populations is known to be affected by pericardiectomy, even long-term post-surgery.^18^

Meanwhile, FAC correlated moderately with CMR-RVEF and was comparable to 3D-RVEF and RV LS. FAC is however geometry and load dependent and does not include the contribution of the RVOT to RV ejection.^19^ In ToF patients with dysfunctional RVOT for instance, FAC will often result in an overestimation of global RV function.^2^ Still, this is a useful conventional parameter to use in an ACHD population and in this study, FAC did not perform inferiorly to 3D-RVEF.

### Advanced echocardiographic parameters

Most RV wall LS values correlated moderately with CMR-RVEF, however multiple wall average values were not superior to LS measurements from the standard apical four-chamber view. While 2D-MPE enables a more global RV deformational analysis to be performed, this appears more pertinent to ACHD with abnormalities of the RVOT, such as ToF. Here, anterior wall deformation is reduced,^9^ with proximity to a dyskinetic RVOT a likely factor following initial repair or subsequent re-intervention.^2^ CMR studies have also demonstrated the presence of fibrosis in adult patients in the surrounding myocardial segments.^20,21^

3D-RVEF correlated less strongly with CMR-RVEF than LS or FAC measurements and underestimated RV volumes and ejection fraction. This has been widely identified in previous 3D-TTE-CMR comparative studies, which report that while volumes generally correlate well, those of 3D-TTE are 20–34% smaller.^1,4,7^ Some studies have however reported better association between respective RVEF measurements.^6,7^ A major limitation of 3D-TTE is the poor visualization of the anterior RV wall. Artefact from the sternum, scar tissue, or intra-cardiac prosthetic material related to previous surgical interventions leads to echo drop out and volume underestimation.^4^ In contrast, CMR is less limited by near field resolution and thus endocardial borders can be better delineated.^2^ Furthermore, in significantly dilated and/or abnormally shaped RVs, 3D-TTE may fail to fully accommodate the entire chamber within the pyramidal dataset. This can also lead to foreshortening of the RV apex and significant underestimation of RV volumes.^2,4^

RV lateral (free) wall and global LS values were significantly greater (i.e. more negative) when measured by CMR-FT than by STE. The poor agreement between values may be due to differences in image processing software and the inability to achieve the same imaging plane. RV LS derived by CMR-FT is however not by definition less useful than STE and has been shown to be an independent predictor of adverse events and mortality.^22,23^ Differences in image resolution between TTE and CMR should also be considered. Spatial resolution is higher in CMR images, enabling more accurate tracking of the RV endocardium than by TTE. On the other hand, temporal resolution in the CMR loops was much lower (30 frames per cardiac cycle) than that recorded for TTE strain analysis (>60 frames per second). Lower temporal resolution results in larger distances covered by the features between frames and requires an enlarged interrogation window, which may decrease accuracy.^24^ Nonetheless, it has been reported that acquisitions at 30 frames per cardiac cycle offer consistent strain assessments in CMR when compared with higher temporal resolutions.^25^ A lower signal to noise ratio in TTE means that some segments of the myocardium may not be adequately imaged throughout the cardiac cycle and require averaging of the measured values to fill these gaps.^24^ Lastly, CMR strain analysis was performed on a different vendor to that of TTE, and significant inter-vendor variability has been previously described for CMR-derived RV LS measurements.^26^

### Clinical impact and future directions

Our findings demonstrate that where feasible, advanced echocardiographic RV functional parameters can be incorporated into routine ACHD follow up, albeit with the awareness of the differences that exist with CMR-derived measurements. The increasing availability of automated RV 3D and strain software modalities on the echo machine will help to facilitate the transition to daily clinical practice.^27,28^ Technological advances may make 3D-STE of the RV feasible and attractive in the coming years. In a recent publication, Moceri *et al.*^29^ demonstrated strain analysis of multiple RV regions derived from one 3D acquisition in congenital and healthy populations. Quantification currently requires much post-processing using custom built programmes and therefore this technique is not yet ready for clinical practice.

### Limitations

The study population suffers from some selection bias as reduced CMR-RVEF was present in only 23 patients. Although low levels of RV dysfunction were unforeseen, a greater proportion of impaired RVs are required to adequately investigate the ability of echocardiographic parameters to identify reduced CMR-RVEF. Due to lower feasibility of 3D-RVEF and multi-RV wall LS indices, evaluation by disease group was deemed insufficient to report. Despite including a relatively large number of ACHD patients undergoing same day TTE and CMR, a larger sample size would therefore be desirable for future studies.

## Conclusion

In ACHD patients, advanced echocardiographic RV functional indices are moderately correlated with reference CMR-derived RV function, although significant differences exist between indices measurable by both modalities. 3D-RVEF, LS, and/or FAC should be used to quantify RV function when image quality is adequate. Multi-RV wall evaluation may only provide additional functional information when notable regional wall motion abnormalities are present.

## Consent

All participants provided written informed consent.

## Data Availability

The data underlying this article will be shared on reasonable request to the corresponding author.

## Lead author biography

Daniel Bowen is a research echocardiographer at Erasmus MC in Rotterdam, The Netherlands. He trained in echocardiography at Royal Papworth Hospital, Cambridge, UK, following completion of an undergraduate degree in Clinical Physiology at Middlesex University, London. He has recently completed his PhD entitled “Novel echocardiographic techniques for the assessment of right and left ventricular function”.
